# 여성 류마티스 관절염 환자의 건강관련 삶의 질 구조모형

**DOI:** 10.4069/kjwhn.2023.06.05

**Published:** 2023-06-30

**Authors:** Bukyung Kim, Mi-Hae Sung

**Affiliations:** 1 College of Nursing, Inje University, Busan, Korea; 1 인제대학교 간호대학; 2 Institute of Health Science, College of Nursing, Inje University, Busan, Korea; 2 인제대학교 간호대학 건강과학연구소

**Keywords:** Health-related quality of life, Psychological resilience, Rheumatoid arthritis, Self-efficacy, Social support, 건강관련 삶의 질, 극복력, 류마티스 관절염, 자기효능감, 사회적 지지

## Abstract

**Purpose:**

This study aimed to construct a structural equation model to explain and predict factors affecting the health-related quality of life (QoL) in female rheumatoid arthritis (RA) patients based on the health-related QoL model by Ferrans et al. (2005) and a literature review.

**Methods:**

Patients (N=243) who were either registered members of an internet cafe composed of patients with RA or rheumatology outpatients at two tertiary general hospitals in Busan, Korea, were recruited via convenience sampling. Data were collected from July 2 to September 9, 2021, and the survey was conducted using a web-based questionnaire. The data were analyzed by SPSS and AMOS 26.0.

**Results:**

The goodness-of-fit statistics of the final model exhibited good results (χ^2^/degree of freedom=2.68, Turker-Lewis index=.94, comparative fit index=.96, standardized root mean-squared residual=.04, root mean- square error of approximation=.08), and 11 out of 14 paths of the model were supported. The squared multiple correlation, which reflected the explanatory power of the environmental characteristics, symptoms, functional status, and perceived health status on health-related QoL, was 80%. In the hypothesis model, 10 paths had significant direct effects, 6 paths had significant indirect effects, and 12 paths had significant total (direct and indirect) effects.

**Conclusion:**

Considering that factors directly affecting the health-related QoL of female patients with RA were social support, symptoms (fatigue and depression), resilience, and perceived health status, and that resilience was the most influential factor, clinicians can encourage resilience. Hence, to improve the health-related QoL of female patients with RA, continuing management is necessary, using various intervention methods that focus on enhancing resilience from the early stage to the end of treatment for RA.

## Introduction

류마티스 관절염(rheumatoid arthritis)은 관절 활막의 지속적인 염증 반응이 특징인 만성 염증성 질환이다[1]. 류마티스 관절염은 전 세계적으로 성인 인구의 약 0.3%–1.0%에서 발생하며, 우리나라에서는 전 인구의 약 1.0%를 차지하고 여성의 유병률이 남성보다 약 3배 정도 높으며 주로 40–70세 사이에 흔하게 나타난다[2]. 류마티스 관절염의 증상은 통증, 부종, 조조강직, 위축과 기형 등의 관절 장애 등이며, 류마티스 관절염 환자들은 피로, 불안, 우울 등 심리적 증상들을 경험한다[2,3]. 특히 류마티스 관절염 환자 중 여성이 남성에 비해 주관적인 통증과 우울이 더 심하게 나타나며[3], 이러한 다양한 신체적·심리적 증상들은 여성 류마티스 관절염 환자의 건강관련 삶의 질(health-related quality of life)을 저하하는 직접적인 요인이다[4]. 류마티스 관절염은 한 사람의 삶을 전반적으로 위협하지만, 주로 일상생활에서 질병으로 인한 신체적, 정서적, 사회적인 영향에 대한 자가 평가를 강조한 개념인 건강관련 삶의 질을 떨어뜨린다[4,5]. 이에 만성질환인 류마티스 관절염 환자들의 치료, 중재를 포함한 질환 관리의 방향을 결정하는 데 건강관련 삶의 질의 고려가 필요하다.

류마티스 관절염 환자를 대상으로 삶의 질의 구조모형을 검증한 국외 연구에서는 삶의 질과 관련된 이론적 기틀을 제시하지 않고 삶의 질에 대한 변수들의 영향력을 분석하고 있으며[6], 국내 연구들에서는 기존에 연구가 되었던 통증, 피로, 그리고 신체적 기능제한 등에 관한 변수만을 다루고 있으므로[4,7] 만성질환인 류마티스 관절염 환자의 신체적·심리적 증상, 기능상태 외에 건강관련 삶의 질에 대해 긍정적인 영향을 미칠 수 있는 요인들을 다각도로 파악하여 건강관련 삶의 질에 미치는 영향을 설명하는 연구가 필요하다. 최근 만성질환자를 대상으로 한 삶의 질 관련 연구를 살펴보면, 자기효능감(self-efficacy) [8], 외상 후 성장[9], 극복력 (resilience) [10] 등 주로 질병을 긍정적으로 수용하며 나아가는 특성에 관한 연구가 진행되고 있다. 이에 류마티스 관절염 환자를 대상으로 심리적인 요소에 기반한 자기효능감과 현재 남아있는 기능의 최적화에 초점을 맞춰 극복력을 건강관련 삶의 질에 관한 영향요인으로 투입하여 건강관련 삶의 질에 미치는 영향을 포괄적으로 파악할 필요가 있다. 또한 객관적인 건강상태가 양호함에도 불구하고 자신의 건강상태를 어떻게 지각하느냐에 따라서 건강상태가 다르게 나타날 수 있으므로 지각된 건강상태(perceived health status)도 중요한 측정지표이다[11]. 그러나, 류마티스 관절염 환자의 건강관련 삶의 질에 미치는 영향요인으로 지각된 건강상태를 고려한 연구는 저조한 실정이다. 그러므로, 여성 류마티스 관절염 환자의 증상과 기능상태가 지각된 건강상태와 건강관련 삶의 질에 영향을 주는 특정요인으로 작용하는지 살펴볼 필요가 있다.

Ferrans 등[12]은 Wilson과 Cleary [13]의 대상자 건강 결과에 대한 개념적 모델(health-related quality of life: a conceptual model of patient outcomes)을 수정하여 모형을 제시하였다. Ferrans 등[12]은 Wilson과 Cleary [13]가 설명하지 않은 개인적, 환경적 특성에 대해 이론적 근거를 제시하여 간호와 건강관리 영역에서 각 구성요소를 적용하기 위한 방안을 설명하고 있다. Ferrans 등[12]의 건강관련 삶의 질 모형을 적용한 연구에서는 자궁암[14], 당뇨병[15], 만성 폐쇄성 폐질환[16]등을 다루며, 주로 만성질환자를 대상으로 한 연구들이 있다. 이에 만성질환인 여성 류마티스 관절염 환자에게도 Ferrans 등[12]의 건강관련 삶의 질 모형을 이론적 기틀로 적용하여 건강관련 삶의 질에 미치는 영향을 설명할 수 있을 것이라 생각한다.

Ferrans 등[12]은 건강관련 삶의 질에 영향을 미치는 요인으로 생물학적 기능, 증상, 기능상태, 일반적 건강 지각 등을 보았으며, 이들은 건강관련 삶의 질에 순차적으로 영향을 미치며, 개인적 특성과 환경적 특성은 이러한 요인들을 통하여 건강관련 삶의 질에 영향을 미치는 것으로 설명하였다. 생물학적 기능은 검사 결과, 신체 사정, 그리고 의학적 진단 같은 지표들을 통해 평가하는데[12], 이들은 중재를 할 수 없는 불가항력적인 요인으로 생물학적 기능은 본 연구의 모형에 포함하지 않고, 질병관련 특성에 따른 건강관련 삶의 질 차이로 분석하였다. 증상은 비정상적인 상태에 대한 신체적, 정서적 혹은 인지적 상태에 대한 대상자의 지각[13]으로 정의하고 있으며, 기능상태는 현재 남아 있는 기능의 최적화에 초점을 맞춰 기능적 용량, 기능적 수행, 기능적 용량 활용 및 기능적 예비력 등의 4가지 차원을 포함한다[17]. 일반적인 건강 지각은 2가지 특징을 가지고 있는데, 모형에서 초기에 제시된 요소들을 통합하는 것과 본질적으로 주관적이라는 특징을 지니고 모형의 최종 구성 요소인 전반적인 삶의 질은 개인이 삶 전체에 대해 얼마나 행복하고 만족하는지를 의미하는 주관적 안녕[13]으로 정의된다. 개인적 특성은 건강 결과에 영향을 미치는 인구통계적, 발달적, 심리적 및 생물학적 요인으로 분류되며, 심리적 요인은 질병, 치료, 행위에 대한 지식, 신념, 태도를 포함하는 인지적 평가로 규명된다[18]. 환경적 특성은 가족, 친구, 의료 서비스 제공자의 영향을 포함한 건강 결과에 대한 대인관계 또는 사회적 영향을 포함한다[12].

이에 본 연구에서 개인적 특성은 심리적인 요소에 기반한 자기효능감을 개념으로 선정하고, 환경적 특성은 사회적 지지(social support)를 선정하였으며, 증상은 피로 및 우울로 선정하였다. 기능상태로는 스트레스의 부정적인 효과를 중재하고 적응을 증진하는 긍정적인 개인의 능력으로 정의되는[19] 극복력을 선정하였으며, 극복력은 질병과 스트레스 요인에 긍정적으로 작용하여 만성질환자의 건강관련 삶의 질 영향요인임이 밝혀졌다[14]. 대부분의 연구[8,20]에서 신체적 기능만을 기능상태로 측정하고 있으며, 심리적 기능에 초점을 맞춘 연구는 거의 없는 실정이다. Ferrans 등[12]은 기능상태를 현재 처해 있는 기능상태의 최적화에 초점을 둔다고 하였으므로 Ferrans 등[12]이 설명한 기능상태 중 심리적 기능상태의 측면에서 극복력을 측정하였다.

**Figure 1.**
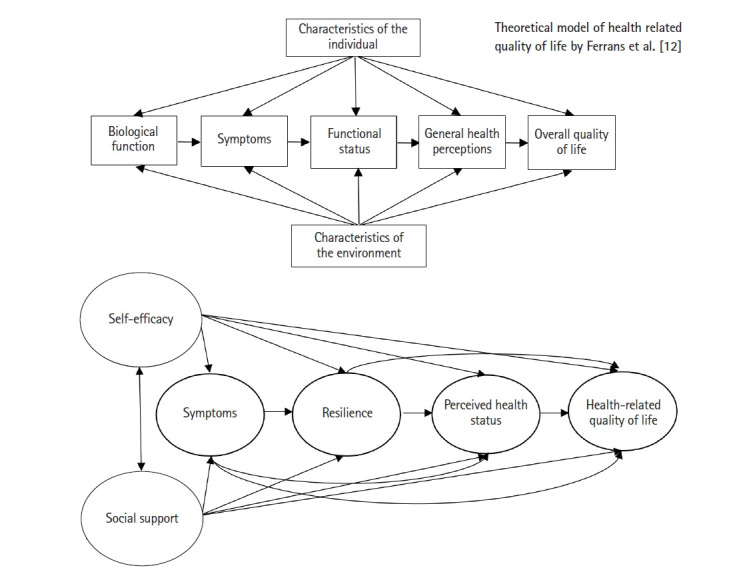
Hypothetical model of this study.

일반적인 건강지각은 지각된 건강상태로 측정하였다. Ferrans 등[12]의 모형에서 생리학적 기능을 제외한 모든 경로를 설정하여 가설적 모형을 구성하였으며, 문헌고찰을 토대로 자기효능감과 사회적 지지의 상관관계인 경로[20,21]와 증상(피로, 우울)과 극복력이 건강관련 삶의 질로 가는 직접적인 경로[8,14,20]와 증상(피로, 우울)이 지각된 건강상태로 가는 직접적인 경로[22]를 추가하였다(Figure 1). 따라서, 자기효능감과 사회적 지지가 서로 영향을 주며, 자기효능감과 사회적 지지는 증상, 극복력, 지각된 건강상태를 거치면서 최종적으로 삶의 질에 영향을 미치고, 증상은 지각된 건강상태와 건강관련 삶의 질에 직접적으로 영향을 미치고, 극복력도 건강관련 삶의 질에 직접적으로 영향을 주는 가설적 모형을 구축하고자 한다. 또한 이를 검증하여 여성 류마티스 관절염 환자들의 건강관련 삶의 질 향상을 위한 간호중재 방향을 설정하는 데 필요한 지식적 토대를 마련하고자 한다.

## Methods

Ethics statement: This study was approved by the Institutional Review Board of Inje University (2021-04-023-002). Informed consent was obtained from the participants.

### 연구 설계

본 연구는 여성 류마티스 관절염 환자의 건강관련 삶의 질에 미치는 영향요인을 예측하기 위하여 Ferrans 등[12]의 건강관련 삶의 질 모형 및 문헌고찰을 바탕으로 가설적 모형을 구축하고, 모형의 적합도 및 가설을 공분산 구조분석으로 검증하는 서술적 인과관계 연구이다. 이 연구는 STROBE (Strengthening the Reporting of Observational Studies in Epidemiology) 보고 지침(http://www.strobe-statement.org)에 따라 기술하였다. 

### 연구 대상

본 연구의 대상자는 류마티스 관절염을 진단받은 여성 환자를 표적 모집단으로, 류마티스 질환 환우회 인터넷 카페(다음 카페: 류마티스를 이기는 사람들, https://cafe.daum.net/rheumatism)에 등록된 회원과 부산시에 소재한 상급 종합병원인 인제대학교병원, 부산대학교병원의 류마티스 내과 외래를 방문한 환자를 근접 모집단으로 한 만 19세 이상인 여성으로 하였으며, 류마티스 내과 전문의로부터 진단검사를 통해 류마티스 관절염으로 진단받고 6개월 이상 통원치료를 받고 있는 자를 대상으로 하였다.

대상자 수 선정을 위해 요구되는 구조방정식 모형에서의 표준 크기는 구조방정식의 최대우도법(maximum likehood estimation)에 적합한 표본 크기로 200명 이상을 요구하는 것에 근거하였다[4,23]. 류마티스 질환 환우회 인터넷 카페에서 모집한 대상자 수인 199명과 상급 종합병원인 인제대학교병원, 부산대학교병원에서 모집한 대상자의 수인 44명으로 총 243명의 대상자 모두를 최종 분석하였다.

### 연구 도구

본 연구에서 사용한 모든 도구들은 사용 전 도구 개발자 및 번안자에게 도구 사용에 대해 허락을 받은 후 구조화된 설문지를 사용하였다.

### 건강관련 삶의 질

세계보건기구에서 개발한 삶의 질 측정도구(World Health Organization Quality of Life Assessment Instrument-Brief, WHOQOL-BREF) [24]를 토대로 Min 등[25]이 번역한 한국판 세계보건기구 삶의 질 단축형 도구(Korean version of WHOQOL-BREF)를 사용하였다. 이 도구는 전반적인 삶의 질과 일반적인 건강의 각각에 대한 1문항, 신체적 건강 영역 7문항, 심리적 영역 6문항, 사회적 관계 영역 3문항, 환경적 영역 8문항 영역으로 구성되어 있으며, 총 26개 문항으로 이루어져 있다. 각 문항은 5점 Likert 척도이고, 3번, 4번, 26번 문항은 부정문항으로 역환산하였다. 본 연구에서는 한국형으로 번안한 Min 등[25]의 scoring 방법을 따라 총점을 구하였으며, 점수 범위는 26–130점으로 점수가 높을수록 삶의 질이 높음을 의미한다. 개발 당시 도구[24]의 신뢰도는 Cronbach’s α=.90이었으며, Min 등[25]의 연구에서는 .89, 본 연구에서는 .93이었다.

### 자기효능감

Chen 등[26]이 개발한 일반적 자기 효능 척도(New General Self-efficacy Scale)를 Cho 등[27]이 번안한 도구를 사용하였다. 이 도구는 총 8개 문항의 5점 Likert 척도로, ‘전혀 그렇지 않다’ 1점에서 ‘매우 그렇다’ 5점으로 이루어져 있다. 점수 범위는 8–40점으로, 점수가 높을수록 자기효능감 정도가 높음을 의미한다. 개발 당시 도구[26]의 신뢰도는 Cronbach’s α=.85였으며, Cho 등[27]의 연구에서 .83, 본 연구에서는 .94였다.

### 사회적 지지

Zimet 등[28]이 개발한 지각된 사회지지 다차원 척도(Multidimensional Scale of Perceived Social Support)를 Shin과 Lee [29]가 번역한 도구를 사용하였다. 이 도구는 가족 지지(4개 문항), 친구 지지(4개 문항), 의미 있는 타인에 의한 특별 지지(4개 문항)의 3가지 하위 영역으로, 총 12개 문항이 각각 ‘전혀 그렇지 않다’ 1점에서 ‘매우 그렇다’ 5점의 5점 Likert 척도로 이루어져 있다. 점수 범위는 12–60점으로 점수가 높을수록 사회적 지지 정도가 높은 것을 의미한다. 개발 당시 도구[28]의 신뢰도는 Cronbach’s α=.85였으며, Shin과 Lee [29]의 연구에서는 .89였고, 본 연구에서는 전체 .95, 가족 지지는 .92, 친구 지지는 .93, 의미 있는 타인에 의한 특별 지지는 .92였다.

### 피로

Schwartz 등[30]이 개발한 fatigue Assessment Inventory를 토대로 Chang [31]이 개발한 다차원 피로 척도(Multidimensional Fatigue Scale)를 사용하였다. 이 도구는 전반적 피로도(8개 문항), 일상생활 기능장애(6개 문항), 상황적 피로(5개 문항)의 총 19문항으로 구성되어 있다. ‘전혀 그렇지 않다’ 1점에서 ‘매우 그렇다’ 5점의 5점 Likert 척도로, 점수 범위는 19–95점으로 점수가 높을수록 피로 정도가 높은 것을 의미한다. 개발 당시 도구[31]의 신뢰도는 Cronbach’s α=.88이었으며, 본 연구에서는 .95였다.

### 우울

Radloff [32]가 개발하고 Eaton 등[33]이 개정한 Center for Epidemiologic Studies Depression Scale -Revised (CESD-R)를 Lee 등[34]이 국내 실정에 맞게 번안한 한국판 역학연구 우울척도 개정판(Korean version of CESD-R)을 사용하였다. 이 도구는 총 20개 문항으로 구성되어 있으며, 5점 Likert 척도로 최근 일주일 간 우울 관련 증상을 얼마나 자주 느끼는지에 따라서 ‘1일 미만’ 0점에서 ‘2주간 거의 매일’ 4점으로 이루어져 있다. 본 연구에서 통계분석을 위해 1–5점 척도로 변환하여 사용하였으며, 점수가 높을수록 우울 정도가 높은 것을 의미한다. 개발 당시 도구[33]의 신뢰도는 Cronbach’s α=.85–.90이었으며, Lee 등[34]의 연구에서는 .98이었다. 타당도 분석 결과 3개(1, 11, 18번 문항)의 요인 부하량이 .50 미만으로 낮아 이를 제외한 총 17개의 문항을 최종 분석에 사용하였고, 본 연구에서의 Cronbach’s α=.94였으며 총 점수 범위는 17–85점이었다.

### 극복력

Wagnild와 Young [19]이 개발한 극복력 척도(Resilience Scale)를 Song [10]이 번안한 도구를 사용하였다. 이 도구는 2개의 하위영역으로 개인의 유능성 17개 문항, 자신과 삶의 수용 8개 문항의 총 25개 문항으로 구성되어 있다. ‘매우 그렇지 않다’ 1점에서 ‘매우 그렇다’ 5점의 5점 Likert 척도로, 점수가 높을수록 극복력이 높음을 의미한다. 개발 당시 도구[19]의 신뢰도는 Cronbach’s α=.88이었으며, Song [10]의 연구에서는 .87이었다. 타당도 분석 결과 2개(11, 20번 문항)의 요인 부하량이 .50 미만으로 낮아 이를 제외한 총 23개의 문항을 최종 분석에 사용하였으며, 총 점수 범위는 23–115점이었다. 본 연구에서의 전체 신뢰도는 Cronbach’s α=.93이었고, 개인의 유능성은 .91, 자신과 삶의 수용은 .84였다.

### 지각된 건강상태

Speake 등[35]이 개발한 지각된 건강상태(Perceived Health Status)를 Hwang [36]이 번안한 도구를 사용하였다. 총 3개 문항으로 구성되어 있으며, ‘매우 나쁘다’ 1점에서 ‘매우 좋다’ 5점의 5점 Likert 척도로 점수가 높을수록 지각된 건강상태 정도가 양호함을 의미하며, 총 점수 범위는 3–15점이다. 개발 당시 도구[35]의 신뢰도는 Cronbach’s α=.85였으며, Hwang [36]의 연구에서는 .85였고, 본 연구에서는 .84였다.

### 자료 수집

자료 수집 기간은 2021년 7월 2일부터 9월 9일까지였으며, 연구자는 류마티스 질환 환우회 인터넷 카페(다음 카페: 류마티스를 이기는 사람들, https://cafe.daum.net/rheumatism)의 여성 류마티스 관절염 환자와 해당 상급 종합병원의 류마티스 내과를 방문한 환자 중 연구대상자의 선정기준에 적합한 대상자를 편의 표집하였다. 류마티스 질환 환우회 인터넷 카페의 운영자에게 메일을 통해 연구에 관련하여 설명하고, 자료 게시에 대한 동의를 얻은 이후 대상자에게 인터넷 설문지의 양식을 사용하였다. 대상자가 설문에 응하고자 하는 경우 모집공고문에 제시된 설문지 링크를 클릭하여 설문 참여 페이지로 넘어가게 하여 연구에 대한 설명과 동의를 받은 후에 자가보고 형식의 설문 조사를 실시하였다. 또한, 해당 상급 종합병원의 부서장에게 허락을 받고 진행하였으며, 류마티스 내과 외래에 대기 중인 환자 중 외래 간호사가 선정기준에 맞는 대상자에게 참여 의사를 물었다. 참여 의사를 밝힌 대상자를 설문지 작성이 가능한 개별적인 공간으로 안내하여 연구자가 선정기준에 적합한 대상자에게 연구에 대한 설명을 하고 동의를 받은 후, 자료를 수집하였다. 도구 이외에 일반적 특성 및 질병 관련 특성과 관련한 연령, 결혼상태, 교육수준, 경제상태, 종교 유무, 체질량지수, 류마티스 관절염으로 진단받은 기간, 류마티스 관절염 외 질병 유무, 류마티스 관절염으로 인한 수술 여부, 류마티스 관절염과 관련한 약물 복용 여부 등을 조사하였다. 설문 작성에 약 15분 내외가 소요되었으며, 연구에 참여한 모든 대상자에게 소정의 답례품을 제공하였다.

### 자료 분석 방법

수집된 자료는 IBM SPSS ver. 28.0과 AMOS 26.0 (IBM Corp., Armonk, NY, USA)을 이용하여 다음과 같이 분석하였다.

• 대상자의 특성 및 측정변수의 서술적 통계는 빈도, 평균, 표준편차 및 백분율 등의 기술통계를 사용하여 분석하였다.

• 대상자의 일반적 특성 및 질병관련 특성에 따른 삶의 질은 independent t-test와 분산분석(analysis of variance)으로 분석하였으며, 사후 검정으로는 Scheffé post hoc test로 검증하였다.

• 대상자의 자기효능감, 사회적 지지, 증상, 극복력, 지각된 건강상태, 건강관련 삶의 질 간의 상관관계 분석은 Pearson correlation coefficient로 분석하였다.

• 측정 모형의 타당성을 평가하기 위해 확인적 요인 분석(confirmatory factor analysis)를 시행하였다.

• 건강관련 삶의 질에 영향을 미치는 요인 간의 직, 간접 경로계수를 산출하기 위해 공분산 구조분석으로 하였으며, 다변량 정규성을 가정하는 최대우도법(maximum likelihood)을 사용하였다.

• 건강관련 삶의 질 가설적 모형에 대한 적합도 검정은 chi-square (χ^2^), χ^2^/degree of freedom (df), Turker-Lewis index (TLI), comparative fit index (CFI), standardized root mean-squared residual (SRMR), root mean-square error of approximation (RMSEA)을 사용하였다.

• 건강관련 삶의 질 가설적 모형의 경로에 대한 유의성은 표준화 계수(standardized coefficient), standard error, critical ratio, *p*값으로 확인하였으며, 내생 변수의 설명력은 다중 상관제곱(squared multiple correlation)으로 평가하였다.

• 건강관련 삶의 질 가설적 모형의 총효과와 직접·간접효과의 통계적 유의성 검정을 위해 붓스트레핑(boostrapping)을 사용하였다.

## Results

### 대상자 특성에 따른 건강관련 삶의 질 차이

대상자의 특성 중 연령은 평균 47.24세로 50세 이상이 41.2% (100명)로 가장 많았고, 결혼상태는 기혼인 군이 74.1% (180명)로 가장 많았다. 대상자의 교육수준은 대졸인 군이 62.6% (152명)로 가장 많았고, 경제상태는 ‘중’으로 응답한 군이 68.7% (167명)로 가장 많았다. 종교는 ‘무’로 응답한 군이 54.3% (132명)이었다. 대상자의 체질량지수는 평균 22.18 kg/m^2^이었고 정상인 군이 53.9% (131명)로 가장 많았으며, 류마티스 관절염으로 진단받은 기간은 10년 이상인 군이 38.7% (94명)로 가장 많았다. 류마티스 관절염 외 다른 질병 유무는 ‘무’로 응답한 군이 53.1% (129명)로 많았으며, 류마티스 관절염으로 인한 수술 여부는 ‘무’로 응답한 군이 85.6% (208명)으로 가장 많았다. 류마티스 관련 약물 복용여부에서 ‘유’로 응답한 군이 97.1% (236명)로 많았다(Table 1).

대상자의 건강관련 삶의 질에 차이를 보이는 특성은 교육수준(F=6.38, *p*=.002), 경제상태(F=20.17, *p*<.001), 류마티스 관절염 외 다른 질병 유무(t=–2.12, *p*=.034), 류마티스 관련 약물 복용 여부(t=–2.81, *p*=.005)였다. 교육수준이 ‘대학원 졸업’인 경우가 ‘고등학교 졸업’ 이하인 경우와 ‘대학교 졸업’인 경우보다 건강관련 삶의 질이 높았으며, 경제상태가 ‘하’인 경우보다 ‘중’인 경우가, ‘중’인 경우보다 ‘상’인 경우가 건강관련 삶의 질 정도가 높았다. 또한 류마티스 관절염 외 다른 질병을 가지고 있지 않은 사람이 류마티스 관절염 외 다른 질병을 가지고 있는 사람보다 건강관련 삶의 질 정도가 높았으며, 류마티스 관련 약물을 복용하지 않는 사람이 복용하는 사람보다 건강관련 삶의 질 정도가 높았다(Table 1).

### 연구변수의 서술적 통계 및 다중공선성 분석

자기효능감 점수는 40점 만점에 평균 26.58점이었으며, 사회적 지지 점수는 60점 만점에 평균 44.81점이었다. 증상을 확인한 피로 점수는 95점 만점에 평균 70.39점이었으며, 우울 점수는 85점 만점에 평균 29.55점이었다. 극복력 점수는 115점 만점에 평균 80.38점이었다. 지각된 건강상태는 15점 만점에 평균 7.92점이었으며, 건강관련 삶의 질은 130점 만점에 평균 80.00점이었다(Table 2). 본 연구 변수의 일변량 정규성을 검증한 결과, 절대값이 왜도 3, 첨도 8을 넘지 않아 단일 변량 정규분포 조건을 만족하였다(Table 2). 본 연구에서의 공차한계 값은 모두 0.1 이상이며, 분산팽창지수 모두 10 이하의 값을 나타내어 측정변수 간의 다중공선성은 존재하지 않는 것으로 나타났다(Table 2).

### 모형의 타당성 검증

#### 확인적 요인 분석

잠재변수에 대한 각 요인들의 요인부하량(standarized factor loading)은 표준화 계수 값이 최소 .50 이상이어야 하며 각 요인부하량이 .50 미만이면 관측 변수를 제거하게 된다[37]. 자기효능감은 단일 변수로 측정하였으며, 사회적 지지는 하위요인인 가족 지지, 친구 지지, 의미 있는 타인에 의한 특별 지지로 분석하였다. 증상은 피로와 우울로 분석하였으며, 극복력은 하위요인인 개인의 유능성과 자신과 삶의 수용으로 분석하였다. 지각된 건강상태는 지각된 건강상태의 건강관련 삶의 질에 대한 영향을 본 선행연구를 토대로 각 문항별로 분석하였다[14]. 건강관련 삶의 질은 선행연구를 토대로[20,25], 영역 점수의 총합인 단일 변수로 측정하였다. 그 외에 각 요인들의 요인부하량을 살펴보면 모두 .50 이상이며, 개념 신뢰도(composite construct reliability)가 기준 값인 0.7 이상, 분산추출지수(average variance extracted)가 기준 값인 0.5 이상으로 집중타당성은 확보되었다(Table 3). 판별 타당도는 상관계수(r)값과 분산추출지수로 확인하며, 각 요인의 상관계수의 제곱 값이 분산추출지수보다 클 경우 잠재 변인 간의 동일한 요인을 측정하는 것을 의미한다. 본 연구에서 건강관련 삶의 질과 자기효능감, 사회적 지지, 극복력, 지각된 건강상태는 정적 상관관계가 있었으며, 건강관련 삶의 질과 증상은 부적 상관관계가 있어 연구자가 선행연구를 토대로 요인 간의 관계를 예측한 방향대로 나타나 법칙 타당도가 확보되었다. 또한 가설적 모형에서 설정한 경로에서 자기효능감과 사회적 지지에 정적 상관관계(r=.50, *p*<.001)가 있는 것으로 나타났다(Table 4).

### 가설 모형의 검증

#### 가설 모형의 적합도 검정

가설적 모형의 적합도를 확인한 결과, χ^2^=109.99 (df=41, *p*<.001), χ^2^/df=2.68, TLI=.94, CFI=.96, SRMR=.04, RMSEA=.08로 나타났다. χ^2^값은 109.99 (*p*<.001)로 기각되어 가설적 모형이 적합하지 않은 것으로 나타났으나[37], 본 연구에서 표본 크기가 200 이상이므로 적합도 판단 시에 χ^2^값에 의존하기보다 다른 적합지수도 함께 고려하여야 한다. 본 연구에서 χ^2^/df는 2–5, TLI는 .90 이상, CFI는 .90 이상, SRMR는 .10 이하, RMSEA는 .10 이하로 모두 좋은 적합도 기준에 부합하여 본 연구의 가설적 모형은 매우 적합한 것으로 나타났다.

#### 가설 모형의 모수 추정

**Figure 2.**
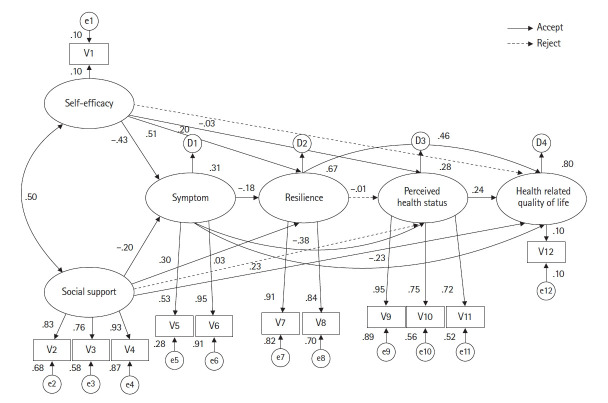
Path diagram of the hypothetical model. V1: self-efficacy, V2: family, V3: friends, V4: significant other, V5: fatigue, V6: depression, V7: personal competence, V8: acceptance of self and life, V9: perceived health status 1, V10: perceived health status 2, V11: perceived health status 3, V12: health-related quality of life.

가설 모형의 모수 추정치 및 통계적 유의성을 검증한 결과, 14개의 경로 중 11개가 통계적으로 유의하였다(Figure 2, Table 5). 자기효능감과 증상(symptom)의 경로계수는 –.43 (*p*<.001), 사회적 지지와 증상의 경로계수는 –.20 (*p*=.005)로 유의한 결과로 나타났다. 증상에 대한 자기효능감과 사회적 지지의 설명력은 31%였다. 자기효능감과 극복력의 경로계수는 .51 (*p*<.001), 사회적 지지와 극복력의 경로계수는 .30 (*p*<.001), 증상과 극복력의 경로계수는 –.18 (*p*=.006)로 유의한 결과로 나타났다. 극복력에 대한 자기효능감, 사회적 지지, 증상의 설명력은 67%였다. 자기효능감과 지각된 건강상태의 경로계수는 .20 (*p*=.045)로 유의한 반면, 사회적 지지와 지각된 건강상태의 경로계수는 .03 (*p*=.751)로 유의하지 않았다. 증상과 지각된 건강상태의 경로계수는 –.38 (*p*<.001)로 유의한 반면, 극복력과 지각된 건강상태의 경로계수는 –.01 (*p*=.955)로 유의하지 않았다. 지각된 건강상태에 대한 자기효능감, 증상의 설명력은 28%였다. 자기효능감과 건강관련 삶의 질의 경로계수는 –.03 (*p*=.569)로 유의하지 않은 반면, 사회적 지지와 건강관련 삶의 질의 경로계수는 .23 (*p*<.001)로 유의하였다. 증상과 건강관련 삶의 질의 경로계수는 –.23 (*p*<.001), 극복력과 건강관련 삶의 질의 경로계수는 .47 (*p*<.001), 지각된 건강상태와 건강관련 삶의 질의 경로계수는 .24 (*p*<.001)로 유의한 결과로 나타났다. 건강관련 삶의 질에 대한 사회적 지지, 증상, 극복력, 지각된 건강상태의 설명력은 80%였다.

#### 가설 모형의 효과 분석

가설적 모형의 잠재 변수 간의 직접효과, 간접효과 및 총 효과는 Table 5에 제시하였다. 증상에 영향을 미치는 직접효과의 경우 자기효능감(β=–.43, *p*=.008), 사회적 지지(β=–.20, *p*=.009)가 통계적으로 유의한 것으로 나타났다. 극복력에 영향을 미치는 직접효과를 살펴보면 자기효능감(β=.51, *p*=.012), 사회적 지지(β=.30, *p*=.004), 증상(β=–.18, *p*=.008)이 통계적으로 유의한 것으로 나타났다. 지각된 건강상태에 영향을 미치는 직접효과의 경우 증상(β=–.38, *p*=.008)이 통계적으로 유의하게 나타났으며, 자기효능감, 사회적 지지 및 극복력은 통계적으로 유의하지 않은 것으로 나타났다. 대상자의 건강관련 삶의 질에 직접효과의 경우 극복력(β=.47, *p*=.009), 지각된 건강상태(β=.24, *p*=.006), 증상(β=–.23, *p*=.009), 사회적 지지(β=.23, *p*=.025)가 통계적으로 유의하게 나타났으며, 자기효능감은 통계적으로 유의하지 않은 것으로 나타났다. 또한 대상자의 건강관련 삶의 질에 간접효과에서는 자기효능감(β=.46, *p*=.019), 사회적 지지(β=.23, *p*=.009), 증상(β=–.18, *p*=.009)이 통계적으로 유의하게 나타났다.

## Discussion

본 연구의 목표는 여성 류마티스 관절염 환자의 건강관련 삶의 질에 영향을 주는 요인을 설명하고 예측하기 위한 가설적 모형을 구축하는 것으로, 본 연구에서 극복력, 지각된 건강상태, 증상(피로, 우울), 사회적 지지의 순으로 건강관련 삶의 질의 80%를 설명하였으며, 자기효능감은 건강관련 삶의 질의 직접적인 영향요인이 아니었다. 류마티스 관절염 환자의 삶의 질 구조모형 연구[4]에서 인구학적 요인, 신체적 요인, 정서적 요인 및 개인적 자원에 의한 설명력이 58.6%이며, 양측 슬관절 전치환술 여성 노인의 구조모형 연구[8]에서 개인적·환경적 특성, 생물학적·생리적 요인, 증상상태, 기능상태 및 건강지각이 건강관련 삶의 질을 62.5%–64.2%로 설명하는 것으로 나타나 본 연구의 설명 모형이 매우 높은 설명력을 지닌 것을 알 수 있다. 이와 같이 연구 모형[4,8]들이 각각 다른 설명력을 지니는 것은 동일한 대상자들에게 모형을 적용한 것이 아니며, 본 연구의 개념적 기틀과는 다르기 때문인 것으로 생각해 볼 수 있다. 본 연구에서는 류마티스 관절염 환자를 대상으로 Ferrans 등[12]의 건강관련 삶의 질 모형에 근거하여 가설적 모형을 세웠으며, 류마티스 관절염 환자의 건강관련 삶의 질에 미치는 요인으로 기존에 연구되지 않았던 극복력과 지각된 건강상태를 함께 투입하여 본 연구의 설명력이 높은 것으로 생각된다.

극복력과 건강관련 삶의 질에 대한 선행연구를 살펴보면, Liu 등[38]에서 류마티스 관절염 환자의 극복력이 정신적 건강관련 삶의 질과 강한 상관관계를 보이며, 삶의 질에 가장 큰 영향요인으로 나타난 것과 본 연구의 결과가 일치한다. 극복력은 심리적 적응력을 증진하여 스트레스를 완화하고, 문제행동을 감소시켜 삶의 질을 증진하는 것으로 보고되고 있다[39]. 따라서, 만성질환인 류마티스 관절염 환자에게서 긍정적 강화요인인 극복력을 향상하는 요인에 대해 파악하고 극복력 향상을 우선적인 중재 목표로 두어 삶의 질의 증진을 위한 전략으로 다루어야 한다.

건강관련 삶의 질에 극복력 다음으로 영향을 미치는 것은 지각된 건강상태로 나타났다. 지각된 건강상태는 임상의가 판단하는 객관적 지수로 반영된 건강수준과 더불어 중요한 요인이므로 여성 류마티스 관절염 환자가 인지하는 건강상태 수준을 사정하는 것은 매우 중요하다. 본 연구에서 지각된 건강상태의 점수는 5점 만점에 평균 평점 2.64점으로, 양측 슬관절 전치환술을 받은 여성 노인의 건강지각이 5점 만점에 3.12점로 나타난 것[8]과 비교하면 본 연구 대상자의 평균 연령이 더 낮았음에도 지각된 건강상태 정도가 낮은 것으로 드러났다. 이러한 결과는 Lee [22]의 연구에서 골관절염 환자와 류마티스 관절염 환자의 지각된 건강상태 정도를 비교하였을 때, 류마티스 관절염 환자의 지각된 건강상태가 더 좋지 않은 것으로 나온 결과를 뒷받침한다. 또한, 본 연구에서 또래와 비교 시 자신의 건강상태가 어떤지 묻는 문항에 대한 점수가 2.16점으로, 다른 문항보다 크게 낮게 나타났다. 이에 류마티스 관절염 환자가 다른 사람과 본인의 건강상태를 비교하지 않고 현재 자신의 건강상태만을 있는 그대로 수용하고 낙관적으로 보도록 돕는 중재전략이 건강관련 삶의 질 향상에 도움이 될 것이다.

본 연구에서 증상은 피로와 우울로 보았으며, 이는 건강관련 삶의 질에 직접효과와 극복력과 지각된 건강상태를 통한 간접효과가 있는 것으로 나타났다. 여성 류마티스 관절염 환자의 피로와 우울 정도가 높을수록 건강관련 삶의 질에 부정적인 영향을 미치는 것을 의미하며, 류마티스 관절염 환자를 대상으로 한 연구[40]에서 피로와 우울은 건강관련 삶의 질에 직접적인 효과가 있으며, 삶의 질에 피로, 불안, 우울, 인지기능 순으로 큰 직접효과를 보인 것과 유사하다. Overman 등[41]은 류마티스 관절염 환자가 호소하는 만성 피로에는 생활습관 개선, 인지행동 요법, 단계별 신체운동 및 수면습관 개선 등과 같은 행동중재를 고려해야 한다고 하였다. 피로는 류마티스 관절염 환자들의 일상생활을 무력하게 만들고 결국 건강관련 삶의 질에 부정적인 영향을 미친다[40]. 따라서 류마티스 관절염 환자들이 호소하는 피로 증상을 가볍게 여기지 않고, 피로의 특성에 대해 면밀히 살펴 이를 적극적으로 관리할 필요가 있다. 우울은 만성 관절염 환자에게서 발생빈도가 높으며, Sheehy 등[42]은 류마티스 관절염을 진단받은 환자에 대한 우울증 선별검사를 추가할 것을 제시하였다. 그러므로, 류마티스 관절염 여성 환자의 건강관련 삶의 질에 대한 개선책을 고려할 때 심리적 건강상태를 반영하는 피로와 우울에 대해 진단 초기부터 면밀히 사정하여 중증의 피로와 우울감을 호소하는 환자들을 선별하고 지속적인 모니터링과 중재관리가 필요하다.

사회적 지지는 건강관련 삶의 질에 직접효과와 증상과 극복력을 통한 간접효과가 있는 것으로 나타났으며, 사회적 지지의 하위영역 중 가족 지지의 점수가 높았는데, 이는 류마티스 관절염 환자의 증상 정도가 심각하여 가족의 적극적인 지원이 이루어지고 있기 때문이라고 예측할 수 있다. 류마티스 관절염 환자의 사회적 지지의 구조적인 측면보다 질적인 측면이 더 중요하며, 류마티스 관절염 환자가 사회적 지지 중 정서적인 지원에 대해 높은 만족도를 느끼는 것으로 보고되고 있어[43], 류마티스 관절염 환자의 지지체계를 강화할 때 가족들이 질 높은 정서적인 지지를 지속적으로 실천할 수 있도록 가족을 대상으로 한 교육 프로그램을 개발 및 적용할 필요가 있다.

본 연구에서 자기효능감은 건강관련 삶의 질에 직접효과는 통계적으로 유의하지 않은 것으로 나타났으나, 증상, 극복력을 통한 간접효과가 있는 것으로 나타났다. 이는 Lee [4]의 연구에서 류마티스 관절염 환자의 건강관련 삶의 질에 자기효능감이 직접효과가 있었던 것과 상이하지만, 선행연구에서 자기효능감은 류마티스 관절염 환자의 정서적 요인과 건강관련 삶의 질 간의 매개변수가 되기도 하고[4] 자기효능감이 증상, 기능상태, 건강지각을 매개로 건강관련 삶의 질에 영향을 미치는 것과 일치한다[8]. 본 연구에서 자기효능감은 여성 류마티스 관절염 환자의 증상(피로, 우울)과 극복력에 가장 큰 영향을 미치는 것으로 나타났는데, 자기효능감이 높을수록 통증과 피로는 감소되고 건강증진행위, 신체활동, 운동 및 치료지시 이행 등이 잘 수행되며[21], 자기효능감 수준을 높이면 질병을 이겨내는 극복력 또한 높은 수준으로 끌어올릴 수 있다[44]. 이러한 결과들은 어려운 상황에서 잠재적으로 이용 가능한 자기효능감이 류마티스 환자의 증상을 완화하고 극복력을 증진하여 건강관련 삶의 질에 영향을 미치는 중요한 특성임을 증명해준다. 자기효능감을 증진하는 것이 만성화된 질병의 증상 관리에 필수적인 역할을 할 수 있을 것으로 생각되며 자기효능감은 건강관련 삶을 설명하는 데 매우 중요한 변수임에도 본 연구에서는 자기효능감이 삶의 질에 직접효과가 없었던 근거를 토대로, 이를 추후 반복 연구를 통해 명확한 인과관계를 확인해 보는 연구들이 필요할 것이다.

본 연구는 Ferrans 등[12]의 이론을 기반으로 여성 류마티스 관절염 환자의 건강관련 삶의 질의 모형을 구축하였다. 이를 통해 간호학적 이론을 생성하여 여성 류마티스 관절염 환자의 건강관련 삶의 질을 향상시키기 위한 다양한 전략을 세울 수 있는 기초자료를 제공하였다는 점에 이 연구의 의의가 있으며, 여성 류마티스 관절염 환자의 삶에 대해 많은 연구자들의 관심을 불러일으키는 계기가 될 것이다. 또한, 본 연구에서 건강관련 삶의 질에 영향을 미친 변수 외에 Ferrans 등[12]의 이론을 근거로 한 다른 변수들을 탐색하고, 추후 여성 류마티스 관절염 환자의 건강관련 삶의 질에 대한 영향 변수로 추가 투입하여 분석하는 연구를 수행할 것을 제언하는 바이다.

## Figures and Tables

**Table 1.** Differences in health-related quality of life according to participants’ characteristics (N=243)

Characteristic	Categories	n (%)	Item	t or F	*p *(Scheffé)
Age (year)	≤39	62 (25.5)	81.06±15.20	1.12	.328
	40–49	81 (33.3)	81.37±16.68		
	≥50	100 (41.2)	78.23±14.55		
Marital status	Single	45 (18.5)	80.77±14.24	1.93	.125
	Married	180 (74.1)	80.32±15.48		
	Separated	14 (5.8)	71.14±15.80		
	Bereaved	4 (1.6)	87.50±21.79		
Education level	≤High school^a^	66 (27.2)	77.39±13.67	6.38	.002
	Bachelor^b^	152 (62.6)	79.50±15.45		(c>a,b)
	≥Master^c^	25 (10.3)	89.88±16.73		
Economic status	High^a^	20 (8.2)	93.80±17.18	20.17	<.001
	Middle^b^	167 (68.7)	81.26±14.21		(a>b>c)
	Low^c^	56 (23.0)	71.28±13.74		
Religion	Yes	111 (45.7)	79.66±14.74	–0.30	.759
	No	132 (54.3)	80.28±16.09		
Body mass index (kg/m^2^)	Underweight (<18.5)	25 (10.3)	79.72±13.34	2.00	.114
	Normal (18.5–22.9)	131 (53.9)	82.10±16.10		
	Overweight (23.0–24.9)	51 (21.0)	77.13±14.77		
	Obese (≥25.0)	36 (14.8)	76.58±14.66		
Time elapsed since receiving the diagnosis (year)	<5	90 (37.0)	80.71±15.20	2.00	.136
	5–9	59 (24.3)	82.61±16.33		
	≥10	94 (38.7)	77.68±14.98		
Comorbidity	Yes	114 (46.9)	77.77±16.18	–2.12	.034
	No	129 (53.1)	81.96±14.58		
Surgery related to RA	Yes	35 (14.4)	77.77±15.95	–0.92	.358
	No	208 (85.6)	80.37±15.38		
Taking medications related to RA	Yes	236 (97.1)	79.52±15.26	–2.81	.005
	No	7 (2.9)	96.00±14.34		

RA: Rheumatoid arthritis.

**Table 2.** Descriptive statistics and multicollinearity of the research variables (N=243)

Characteristics	Categories	Item	Possible range	Skewness	Kurtosis	Tolerance	VIF
Self-efficacy	Total	26.58±6.62	8–40	–0.49	–0.26	0.41	2.40
Social support	Total	44.81±9.63	12–60	–0.44	–0.11	-	-
	Family	15.36±3.57	4–20	–0.59	–0.25	0.35	2.83
	Friends	14.10±3.79	4–20	–0.37	–0.34	0.43	2.29
	Significant other	15.35±3.45	4–20	–0.60	0.01	0.26	3.76
Symptoms	Total	99.95±22.90	36–180	0.66	0.81	-	-
	Fatigue	70.39±13.31	19–95	–0.45	0.27	0.70	1.41
	Depression	29.55±13.12	17–85	1.75	3.41	0.51	1.95
Resilience	Total	80.38±13.27	23–115	–0.24	0.25	–	–
	Personal competence	57.19±9.38	16–80	–0.20	0.19	0.29	3.41
	Acceptance of self and life	23.90±4.50	7–35	–0.22	0.69	0.37	2.70
Perceived health status	Total	7.92±2.51	3–15	0.22	–0.55	-	-
	1^†^	2.79±0.95	1–5	0.19	–0.56	0.33	3.01
	2^†^	2.98±1.05	1–5	0.07	–0.88	0.45	2.18
	3^†^	2.16±0.90	1–5	0.51	–0.27	0.48	2.05
Health-related quality of life	Total	80.00±15.46	26–130	0.07	–0.03	-	-

VIF: Variance inflation factor.

† Indicates each item of perceived health status.

**Table 3.** Factor loading in confirmatory factor analysis (N=243)

Characteristic	Categories	Β	SE	CCR	AVE
Self-efficacy	-	-	-	-	-
Social support	Family	.81	.27	.90	.75
	Friends	.76	.38		
	Significant other	.95	.07		
Symptoms	Fatigue	.54	.34	.83	.73
	Depression	.92	.09		
Resilience	Personal competence	.86	.09	.75	.60
	Acceptance of self and life	.89	.09		
Perceived health status	1^†^	.94	.10	.86	.67
	2^†^	.76	.46		
	3^†^	.72	.39		

AVE: Average variance extracted; CCR: composite construct reliability.

† Indicates each item of perceived health status.

**Table 4.** Correlations between research variables (N=243)

Research variable	r (*p*)					
	V1	V2	V3	V4	V5
V1	1				
V2	.50 (<.001)	1			
V3	–.47 (<.001)	–.34 (<.001)	1		
V4	.68 (<.001)	.57 (<.001)	–.45 (<.001)	1	
V5	.39 (<.001)	.26 (<.001)	–.46 (<.001)	.36 (<.001)	1

V1: Self-efficacy, V2: social support, V3: symptom, V4: resilience, V5: perceived health status, V6: health-related quality of life.

**Table 5.** Standardized direct, indirect, and total effects of the hypothetical model (N=243)

Endogenous variable	Exogenous variable	SE	CR (*p*)	SMC	β (*p*)		
					Direct effect	Indirect effect	Total effect
Symptom	← Self-efficacy	.06	–.6.38 (<.001)	.31	–.43 (.008)		–.43 (.008)
	← Social support	.06	–2.83 (.005)		–.20 (.009)		–.20 (.009)
Resilience	← Self-efficacy	.04	8.42 (<.001)	.67	.51 (.012)	.08 (.004)	.58 (.010)
	← Social support	.04	5.13 (<.001)		.30 (.004)	.04 (.002)	.33 (.007)
	←Symptoms	.05	–2.76 (.006)		–.18 (.008)		–.18 (.008)
Perceived health status	← Self-efficacy	.08	2.00 (.045)	.28	.20 (.051)	.16 (.046)	.36 (.009)
	← Social support	.07	.032 (.751)		.03 (.715)	.07 (.147)	.10 (.172)
	←Symptoms	.09	–3.69 (<.001)		–.38 (.008)	.001 (.866)	–.38 (.012)
	← Resilience	.15	–0.06 (.955)		–.01 (.923)		–.01 (.923)
Health-related quality of life	← Self-efficacy	.04	–0.57 (.569)	.80	–.03 (.628)	.46 (.019)	.43 (.012)
	← Social support	.04	4.87 (<.001)		.23 (.025)	.23 (.009)	.46 (.012)
	←Symptoms	.05	–3.66 (<.001)		–.23 (.009)	–.18 (.009)	–.41 (.011)
	← Resilience	.09	6.13 (<.001)		.47 (.009)	–.002 (.943)	.46 (.007)
	←Perceived health status	.04	5.34 (<.001)		.24 (.006)		.24 (.006)

CR: Critical ratio; SMC: squared multiple correlation.
